# Two Case Reports of Familial Chylomicronemia Syndrome

**DOI:** 10.1155/2012/384719

**Published:** 2012-01-23

**Authors:** Yan-Hui Chen, Zhong-Ling Ke, Yan-Xia Wang, Yong Wang, Yong-Zhi Zheng

**Affiliations:** Department of Paediatrics, The Affiliated Union Hospital, Fujian Medical University, Fuzhou, Fujian 350001, China

## Abstract

Familial chylomicronemia is a rare autosomal recessive disorder which is also called Hyperlipoproteinemia type I. Here we report two cases with this rare disorder that were admitted to our hospital in recent years.

## 1. Case Reports

**Case A**

A 55-day-old boy, G_2_P_2_, who was delivered by cesarean section in full-term pregnancy and was breast-fed, presented at 30 days of age with a short history of brown looser stools, two or three times a day, medium in amount. When he was 40 days old, the baby's face became pale without any petechiae or ecchymoses on skin, and the color of the feces became deeper and deeper till it became completely dark. The local hospital found that the blood of the baby is pink; they were confused and suggested that he be admitted to our hospital. Physical examination revealed a well-developing baby, with a severe anemia appearance. The head circumference of the baby was 40 cm, and the weight of the baby was 5.1 kg. There were two yellow small ulcers in his palate. His abdomen was soft, and the edge of the liver was 2 cm below the right costal margin, while the spleen was palpable 1 cm below the left costal margin. Both the liver and the spleen were soft and smooth. The limbs were normal. There was not any family history of the hyperlipidemia, xanthoma, or pancreatitis. Fasting serum lipids, which included triglyceride (TG) and cholesterol (CHOL) of the baby's parent, were normal. Laboratory examinations include the baby blood routine testing which showed that the hemoglobin (Hb) cannot be tested, while it included red blood cell counts (RBCs) of 1.46 × 10^12^ L^−1^, white blood cell counts (WBCs) of 26.2 × 10^9^ L^−1^, platelet counts of 349 × 10^9^ L^−1^, and the percentage of the neutrophilic segmented granulocyte being 86.0%, the percentage of lymphocyte 2.7%, hematocrit (HCT) 15.4%, and mean corpuscular volume (MCV) 105.5 fL. The coagulation function was normal. The fecal occult blood test was positive. The liver function test showed total protein (TP) 43.0 g·L^−1^, albumin (ALB) 26.0 g·L^−1^CHOL 17.51 mmol·L^−1^, TG 209.00 mmol·L^−1^, high-density lipoprotein cholesterol (HDL-C) 2.08 mmol·L^−1^, low-density lipoprotein cholesterol (LDL-C) 0.25 mmol·L^−1^, apolipoprotein A1 (APOA1) 0.72 g·L^−1^, apolipoprotein B (APOB) 0.50 g·L^−1^, and lipoprotein a (Lp a) 605 mg·L^−1^. The marrow smear showed active proliferation, the ratio of myeloid was 58.5%, and the ratio of erythroid cells was 8.00%. The form and proportion of cells in the different phases were normal and the myeloid proliferates actively while the erythroid hyperplasia was depressed. The size, shape, and color of the mature red blood cell, together with the proportion and form of the lymphocytes and monocytes, were normal. The platelet was commonly visible, and Niemann-Pick histiocyte accounts for 1.5% of the cell in the marrow, whose cells were huge with a single nucleus and 2~3 nucleosome in the plasma, and the plasma of the cells was rich and full of empty bubbles. Fundus examination did not reveal any lipid retinopathy (at which the TG of the baby was lower than 19.87 mmol·L^−1^). The fasting blood of the baby, which was pink, formed cream on the surface after placing overnight in 4°C. Familial chylomicronemia syndrome was diagnosed, based on the information above. The baby was treated by blood transfusion, hemostasis, and some other treatments, but without any drug to reduce the lipids, and was fed with skimmed milk. Four days later, the baby's blood was tested again. The results showed the following: CHOL 32.64 mmol×L^−1^, TG 19.87 mmol×L^−1^, HDL-C 2.13 mmol×L^−1^, LDL-C 0.71 mmol×L^−1^, APOA1 0.77 g×L^−1^, APOB 2.62 g×L^−1^, and Lp a 299 mg×L^−1^. From the fifth day, we supplied the baby with sweet water for 48 hours. At the seventh day, blood test was taken once again; the report showed the following: CHOL 7.00 mmol·L^−1^, TG 6.90 mmol·L^−1^, HDL-C 0.93 mmol·L^−1^, LDL-C 6.61 mmol·L^−1^, APOA1 0.68 g·L^−1^, APOB 0.65 g·L^−1^, and Lp a 30 mg·L^−1^. The baby was followed up for 4 months in the outpatients. With a diet of low fat but trace elements and vitamins added, the TG was controlled in a good level, and the baby was developing well in regard to stature, weight, and mind.

**Case B**

A 60-day-old boy, G_1_P_1_, whose birth weight is 3.45 kg, was easily delivered and breast-fed. When he was 40 days old, the baby face looked pale, and it went more and more pale. At the age of 50 days, the baby showed a dark green mushy stool with a little blood, three or four times a day, medium in amount, accompanied with cough and polypnea. The baby was admitted to the local hospital, and the blood was found to be pink; for this reason the hemoglobin and blood type could not be tested. The baby was transfused with the washed red blood cells of O type and was given antibiotics; after these arrangements, the baby's face turned red and the polypnea was gone. In order to get further diagnosis, his parent brought him to our hospital. We did a careful physical examination to the baby. The baby was developing well, with a medium anemia appearance. His head circumference was of 38 cm, and his weight was 5 kg. The abdomen was soft, and the edge of the liver was 4 cm below the right costal margin, while the spleen was palpable and 3 cm below the left costal margin. Both the liver and spleen were soft and smooth. The limbs were normal. There was not any family history of the hyperlipidemia, xanthoma, or pancreatitis. Fasting serum lipids including triglyceride (TG) and cholesterol (CHOL) of the baby's parent were normal. Laboratory examinations were as follows: Hb could not be tested, RBC 4.01 × 10^12^ L^−1^, WBC 13.8 × 10^9^ L^−1^, N 27.3%, L 63.1%, PLT 486 × 10^9^ L^−1^, HCT 36.8%, MCV 92.0 fL, reticulocyte 0.8%, and the shape and colour of the RBC were normal. The fecal occult blood test was positive. The function of liver and kidney was normal: CHOL 8.64 mmol·L^−1^, TG 90.11 mmol·L^−1^. The fasting serum was milky white. The diagnosis of primaryhyperlipoproteinemia was made. The treatment of the baby included feeding with low-fat milk and supportive care to the baby; afterwards the baby did improve. The baby was followed up for 4 years, having a persistent low-fat diet, and TG was controlled to normal level, and the child is in good nutrition state and developing well.

## 2. Discussion

Familial chylomicronemia syndrome is a rare disorder of lipoprotein metabolism due to familial lipoprotein lipase (LPL) or apolipoprotein C-II deficiency or the presence of inhibitors to lipoprotein lipase [1]. It is characterized by marked elevation of triglyceride and chylomicron levels, resulting in lipemic plasma and recurrent attacks of acute pancreatitis, eruptive xanthoma, hepatosplenomegaly, and lipemia retinalis. The onset of familial LPL deficiency is usually at the age of 10, but 25% of cases occur during infancy. Eruptive xanthomas occurs when serum triglyceride level exceeds 2000 mg/dL and is characterized by asymptomatic, evanescent, yellowish, grouped papules over the buttocks, shoulders, and extensors of limbs. The xanthomas will disappear in a few weeks or a few months after the TG dropping to the low level. Usually, the patient suffers from abdominal pain or pancreatitis from time to time when they are children and often becomes worse after taking fat meal. Because of the disturbance of the lipids and inhibitant in the circulation, the amylase in the serum and urine may turn out to be normal, so it is not easy to diagnose pancreatitis. Secondary complications include diabetes mellitus, steatorrhea, and pancreatic calcification which developed by middle age, while accelerated atherosclerosis and increased risk of coronary artery disease are uncommon [2]. Wilson et al. [3] reported two cases who were of lipid deposition within the central nervous system presented with severe hyperchylomicronemia in early infancy; then, the development of the nervous system was influenced. 

 There is not special drug for these patients, the dietary modification plays a key role in the management of this disease. Concerning the diet, fat should be restricted to <20 g/day and <15% of the total caloric intake, so that triglyceride levels are maintained below 1500 mg/d, and the TG level should be kept below 16.94 mmol·L^−1^. Medium-chain triglycerides are the preferred source of dietary fat, but it should not be over 0.5 g·kg^−1^·d^−1^. Sticking to the fat-restricted diet for a long time, the abdominal pain will be relieved, or even disappear, and liver and spleen will become normal. The coronary artery disease will not be complication, and they can live as long as the others. The two babies in our paper both took the treatment principle as above and got very good results. Nowadays, the key point in treating this disease is to dignose early and to limit the diet; then good results can be obtained.
